# Research trends and hotspots of electroacupuncture for depression: a bibliometric and visual analysis (2005–2025)

**DOI:** 10.3389/fpsyt.2026.1749844

**Published:** 2026-04-28

**Authors:** Xuyang Feng, Qiuxuan Wang, Xi Wu

**Affiliations:** 1 The Second Clinical Medical College, Beijing University of Chinese Medicine, Beijing, China; 2 Dongfang Hospital, Beijing University of Chinese Medicine, Beijing, China; 3 First Teaching Hospital of Tianjin University of Traditional Chinese Medicine, Tianjin, China; 4 National Clinical Research Center for Chinese Medicine, Tianjin, China; 5 Tianjin University of Traditional Chinese Medicine, Tianjin, China

**Keywords:** bibliometric analysis, depression, electroacupuncture, neural mechanism, research trends

## Abstract

**Background:**

Depression is one of the most prevalent mental illnesses worldwide, significantly impairing patients’ quality of life. As a modernized form of acupuncture, electroacupuncture (EA) has been widely applied as an adjunctive therapy for depression. In recent years, both clinical and mechanistic studies on EA have grown rapidly; however, the evidence remains fragmented, and there is still a lack of comprehensive bibliometric and visual analyses summarizing the research hotspots and developmental trends in this field. Therefore, this study aimed to systematically summarize the international research trends, thematic hotspots, and major mechanism-related research themes of electroacupuncture for depression from 2005 to 2025.

**Methods:**

This study was based on the Web of Science Core Collection (WoSCC), which was used to retrieve publications on electroacupuncture for depression from 2005 to 2025. Bibliometric analyses were performed using SCImago Graphica, CiteSpace, and VOSviewer, covering publication trends, countries/regions, institutions, authors, journals, references, and keywords. In addition, PubMed was included as a supplementary database for comparative analysis, primarily to examine keyword distributions and research themes across databases.

**Results:**

A total of 317 studies were included. The annual number of publications increased significantly after 2017. China emerged as the leading contributor and occupied a central position in the international collaboration network. Shanghai University of Traditional Chinese Medicine and author Lao Lixing were identified as major contributors. High-frequency keywords included “acupuncture, ” “depression, ” “anxiety, ” and “electroacupuncture.” The research focus has shifted from clinical outcomes such as “randomized controlled trial, ” “efficacy, ” and “quality of life” to mechanistic aspects, including “neurogenesis, ” “synaptic plasticity, ” “prefrontal cortex, ” “inflammation, ” and “fMRI.” Co-citation and burst analyses revealed that the BDNF/TrkB signaling pathway, NLRP3 inflammasome, prefrontal cortex–amygdala circuit, and HPA axis were major mechanism-related research themes in this field.

**Conclusions:**

This study presents the first systematic bibliometric and visual analysis of publications on electroacupuncture for depression from 2005 to 2025, providing an overview of research trends and thematic developments, and using PubMed as a complementary database. The results indicate that research in this field is shifting from clinically oriented studies toward increasing attention to neurobiological mechanisms, with a growing trend of interdisciplinary integration. Future studies should emphasize high-quality randomized controlled trials, objective biomarker application, and international multicenter collaboration to enhance the research quality and international influence of electroacupuncture in the management of depressive disorders.

## 1 Introduction

Depression represents a major public health burden worldwide, particularly among individuals with chronic diseases and older adults, significantly impairing recovery and quality of life. Although pharmacological treatment remains the primary approach, its limitations—such as delayed onset, poor adherence, and adverse side effects—have become increasingly evident, leading to growing attention on non-pharmacological interventions as complementary or alternative strategies for managing emotional disorders (1–4).

Electroacupuncture (EA), which combines traditional acupuncture with modern electrical stimulation, has emerged as a promising non-pharmacological treatment. Clinical trials and meta-analyses have shown that EA effectively alleviates depressive symptoms, thereby improving patients’ emotional well-being and quality of life, either as a standalone therapy or in combination with antidepressant medications (5–8). Furthermore, previous studies have suggested that EA exerts antidepressant effects by modulating multiple neurobiological pathways, including enhancing brain-derived neurotrophic factor (BDNF) signaling, regulating the hypothalamic-pituitary-adrenal (HPA) axis, attenuating ferroptosis, and inhibiting neuroinflammation. Recent fMRI studies further indicate that EA improves emotion regulation dysfunction by modulating functional connectivity in the prefrontal–amygdala–hippocampal circuit. Although research on EA for depression has expanded rapidly in clinical and mechanistic domains, existing evidence remains fragmented, and a systematic bibliometric and visual analysis of its research landscape, mechanistic focuses, and developmental trends is still lacking (9–13).

Bibliometrics is a quantitative method used to identify research hotspots and development trends in specific fields. In recent years, bibliometric methods have been widely applied to electroacupuncture research for stroke, pain, functional dyspepsia, and other conditions (14–16).

This study presents the first systematic bibliometric and visual analysis of publications on electroacupuncture for depression from 2005 to 2025. The Web of Science Core Collection (WoSCC) was used as the primary data source, with PubMed employed as a complementary database for comparative analysis. Furthermore, this study integrates bibliometric analysis with neurobiological mechanism exploration, aiming to provide a more comprehensive perspective on the field and to offer both theoretical and practical references for future research and clinical translation.

## 2 Methods

### 2.1 Ethical statement

This study was based on a secondary analysis of previously published literature and did not involve any identifiable personal data; therefore, ethical approval was not required.

### 2.2 Data source and search strategy

On September 1, 2025, the WoSCC was used as the primary data source to systematically retrieve publications on electroacupuncture for depression published between 2005 and 2025. The detailed search strategy is presented in **Table 1**.

**Table 1:** Search strategy used in the web of science core collection.

Set	Result	Search query
#1	773, 266	TS=(“depression”OR”depressive disorder”OR”major depressive disorder”OR”depressive symptom”OR”mood disorder”)
#2	8, 192	TS=(“electroacupuncture”OR”electro-acupuncture”OR”electro acupuncture”OR”electrical acupuncture”)
#3	599	Indexes=WoS Core Collection Editions=SCI-Expanded-1900-present;SSCI-1998-present; AHCI-1998-present Document Types=Article, Review Article Timespan=2005-2025 #1AND#2

To enhance the rigor and comprehensiveness of the study and to minimize the limitations associated with reliance on a single database, PubMed was additionally included as a supplementary database for comparative analysis. The corresponding search strategy is provided in **Table 2**.

**Table 2:** Search strategy used in the PubMed.

Set	Result	Search query
#1	218, 437	“Depression”[MeSH Terms] OR “depressive symptoms”[Title/Abstract] OR “depressive symptom”[Title/Abstract] OR “symptom depressive”[Title/Abstract] OR “emotional depression”[Title/Abstract] OR “depression emotional”[Title/Abstract]
#2	8, 895	“Electroacupuncture”[MeSH Terms] OR “Electroacupuncture”[Title/Abstract] OR “electrical acupuncture”[Title/Abstract]
#3	198	Timespan=2005-2025 #1 AND #2 AND #3

All retrieved records were exported in plain text format for subsequent bibliometric analysis.

### 2.3 Inclusion and exclusion criteria

Inclusion criteria were as follows: (1) Publications between January 2005 and September 2025; (2) Article types limited to original research articles and review articles; (3) Studies related to electroacupuncture for the treatment or intervention of depression.

Exclusion criteria were as follows: (1) Non-English publications; (2) Articles with unavailable full text or incomplete data; (3) Studies with low relevance to the research topic (e.g., acupuncture studies focusing primarily on other psychiatric disorders such as bipolar disorder or post-traumatic stress disorder); (4) Publications other than original research articles and reviews (e.g., conference abstracts, editorials, and letters).

### 2.4 Study selection

**Figure 1:**
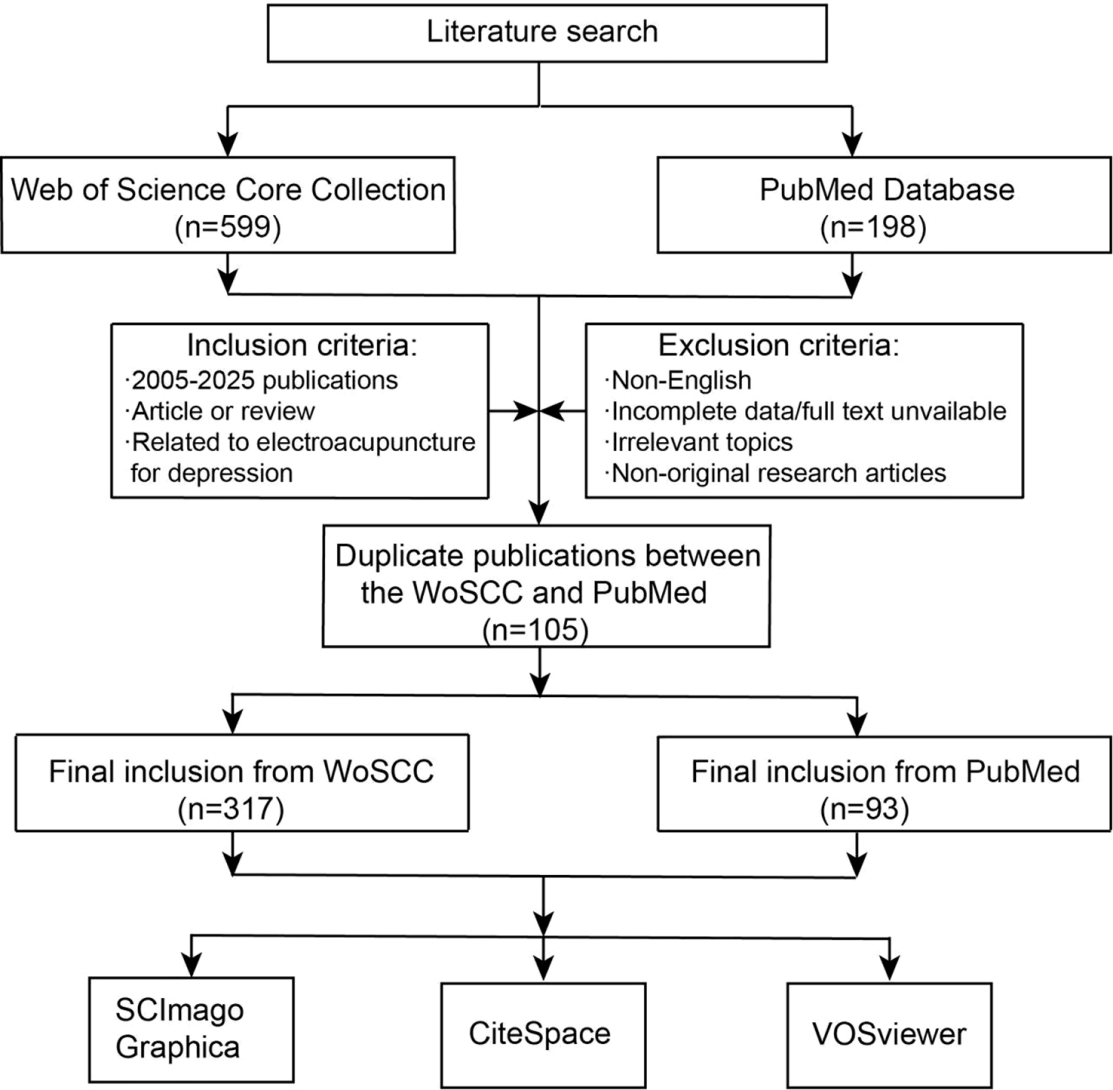
Flowchart of literature search and study selection.

A total of 599 records were initially retrieved from the WoSCC database. After screening based on the predefined inclusion and exclusion criteria, 282 records were excluded, and 317 studies were ultimately included in the final analysis. A total of 198 records were identified from PubMed. After removing 105 duplicate records overlapping with WoSCC using Zotero reference management software, 93 studies were retained for supplementary analysis. All screening and data extraction procedures were independently conducted by two investigators (Xuyang Feng and Xi Wu). Any discrepancies were resolved through discussion or consultation with a third reviewer (Qiuxuan Wang). The study selection process is illustrated in **Figure 1**, and the PRISMA flow diagram is provided in **Supplementary Material 1**.

### 2.5 Data analysis

Bibliometric and visual analyses were performed using VOSviewer (version 1.6.20) and CiteSpace (version 6.4.R1). SCImago Graphica *Beta* 1.0.51was used for geographic visualization and collaboration mapping.

VOSviewer, developed by Nees Jan van Eck and Ludo Waltman from Leiden University, is a widely used tool for bibliometric visualization (17). In this study, VOSviewer was applied to construct and visualize co-authorship networks, co-citation networks, and keyword co-occurrence networks, thereby revealing patterns of academic collaboration and thematic relationships. In VOSviewer, the full counting method was applied for data normalization. The minimum thresholds were set to 2 for countries/regions and institutions, and 3 for authors, journals, references, and keywords.

CiteSpace, developed by Professor Chaomei Chen at Drexel University, was used to explore the evolution of scientific knowledge, research frontiers, and emerging trends in the field (18). In this study, CiteSpace was applied to detect burst keywords and analyze the temporal evolution of research topics. The time slicing was set to one year per slice. The g-index (k = 25) was used as the selection criterion. The link retaining factor (LRF) was set to 2.5, the look-back years (L/N) to 10, and the burst detection threshold (e) to 1.0. Pathfinder was applied for network pruning in all analyses.

### 2.6 Data cleaning and disambiguation

To improve the accuracy and reliability of the bibliometric analysis, data cleaning and disambiguation were performed prior to analysis. Variants of author names, institutional names, and country names were standardized to ensure consistency. Keyword normalization was conducted by merging synonyms, singular and plural forms, and different spelling variants (e.g., “electro-acupuncture” and “electroacupuncture”). The merging of keywords was based on semantic similarity and manual judgment, with reference to the original indexing information in the databases. For entries with ambiguity, two investigators (Xuyang Feng and Xi Wu) independently reviewed and reached a consensus.

## 3 Results

### 3.1 Annual publication trends

**Figure 2:**
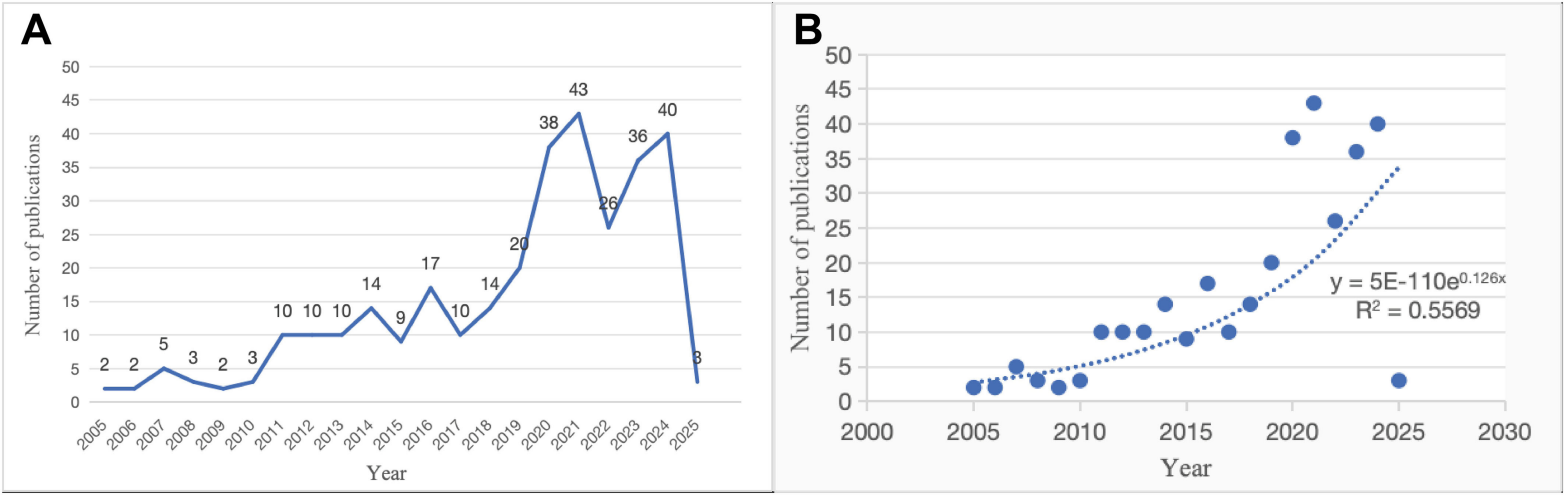
**(A)** Annual number of publications on electroacupuncture for depression from 2005 to 2025. **(B)** Exponential growth fitting curve of publication trends.

From 2005 to 2010, the annual number of publications remained relatively low, averaging 2 to 5 articles per year. Since 2011, the number of publications has shown an upward trend, with notable peaks observed in 2016 (17 articles) and 2021 (43 articles), reflecting growing research interest in this domain. After 2020, the annual publication output has generally remained high, while the decrease in 2025 may be attributed to partial indexing at the time of data retrieval (**Figure 2A**). In addition, curve fitting results (y = 5E-110e^0.126x with R² = 0.5569) indicate a modest model fit (**Figure 2B**).

### 3.2 Country/region analysis

**Figure 3:**
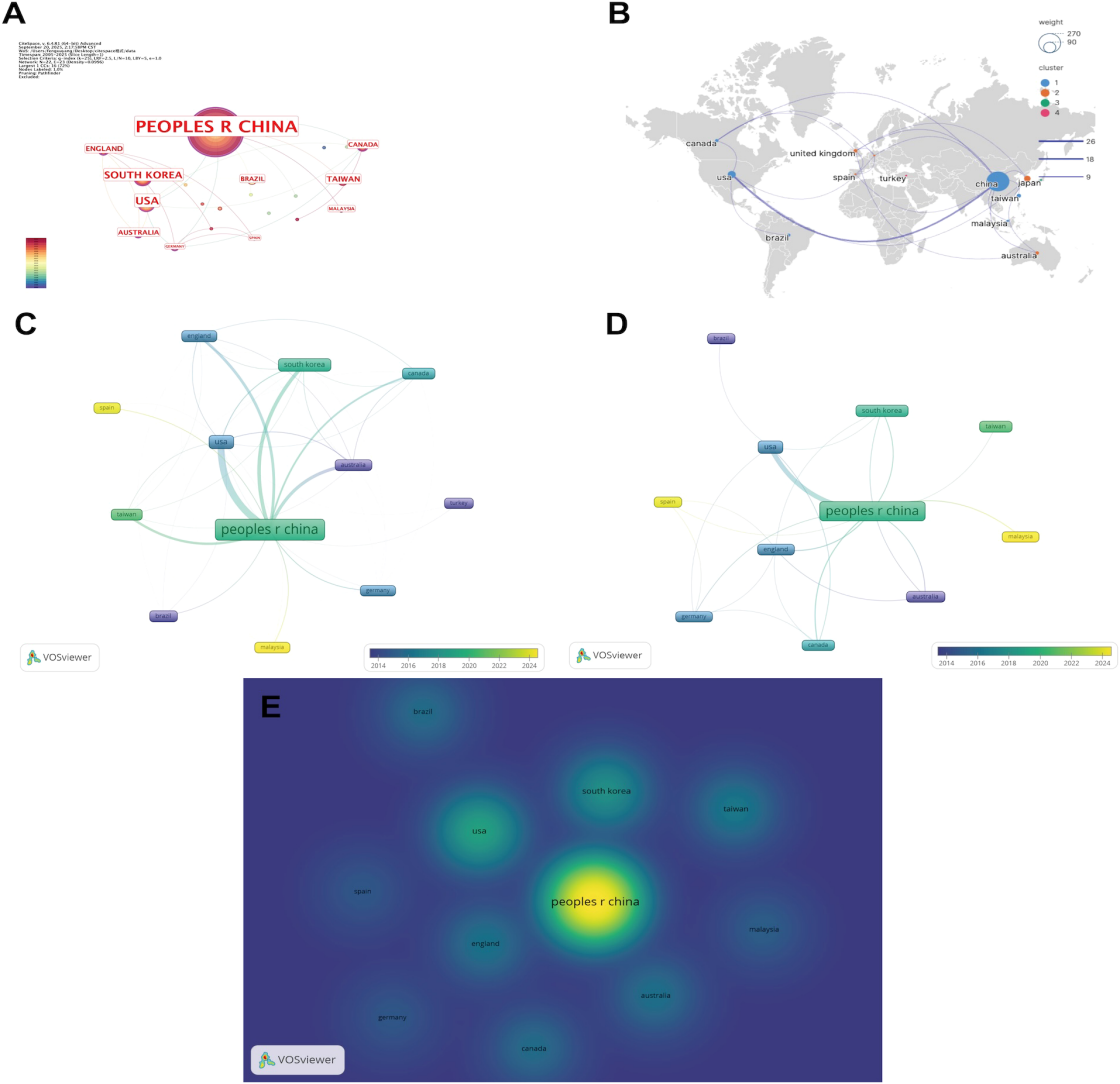
Country/region analysis. **(A)** Visualization of the country/region collaboration network. **(B)** Global geographic distribution of collaborations. **(C)** Overlay visualization of research activity across countries/regions. **(D)** Overlay visualization of citation impact by country/region. **(E)** Density visualization of research intensity by country/region. (Node size represents the number of publications, with larger nodes indicating higher publication output. Node color denotes the average publication year, with warmer colors corresponding to more recent years. The thickness of the connecting lines reflects the strength of collaboration, with thicker lines indicating stronger collaborative relationships. Notably, in **Figure 3B**, node colors represent cluster membership (Clusters 1–4 are distinguished by different colors), with countries/regions within the same cluster forming stable collaborative groups. In **Figure 3E**, warmer colors indicate higher research intensity in the corresponding countries/regions. China, People’s R China: People’s Republic of China. USA, United States of America; UK, England, The United Kingdom of Great Britain and Northern Ireland).

The country/region collaboration network comprised 22 nodes and 23 links (**Figure 3A**). China ranked first in publication volume with 261 articles (82.3%), followed by the United States (33 articles, 10.4%) and South Korea (21 articles, 6.6%). In the collaborative network, China occupied a central hub position, with the most prominent collaboration observed between China and the United States, indicating a high frequency of bilateral cooperation (**Figure 3B**).

The overlay visualization showed that China maintained high research activity over the past five years. The United States, United Kingdom, and Germany were among the earliest countries to engage in this field, while Malaysia and Spain have shown a marked increase in recent activity (**Figures 3C, D**).

The geographic heatmap indicated that research activity was primarily concentrated in China, followed by the United States and South Korea, underscoring the leading role of East Asia and North America in this field (**Figure 3E**).

**Table 3** lists the top 13 most productive countries/regions. Although China had the highest number of publications, its average citation per article was 19.9, which was lower than that of the United Kingdom (51.1) and Australia (70.1). Notably, Germany published only two papers, yet achieved the highest average citation rate of 103 per article, suggesting substantial impact per publication.

**Table 3:** Top 13 most productive countries/regions.

Rank	Country	Articles	Citations	Citations/article
1	China	261	5184	19.9
2	USA	33	1221	37.0
3	South Korea	21	594	28.3
4	Taiwan	10	252	25.2
5	UK	8	409	51.1
6	Australia	7	491	70.1
7	Canada	6	286	47.7
8	Brazil	6	121	20.2
9	Malaysia	3	24	8.0
10	Germany	2	206	103
11	Spain	2	49	24.5
12	Japan	2	20	10.0
13	Turkey	2	29	14.5

### 3.3 Institutional collaboration analysis

**Figure 4:**
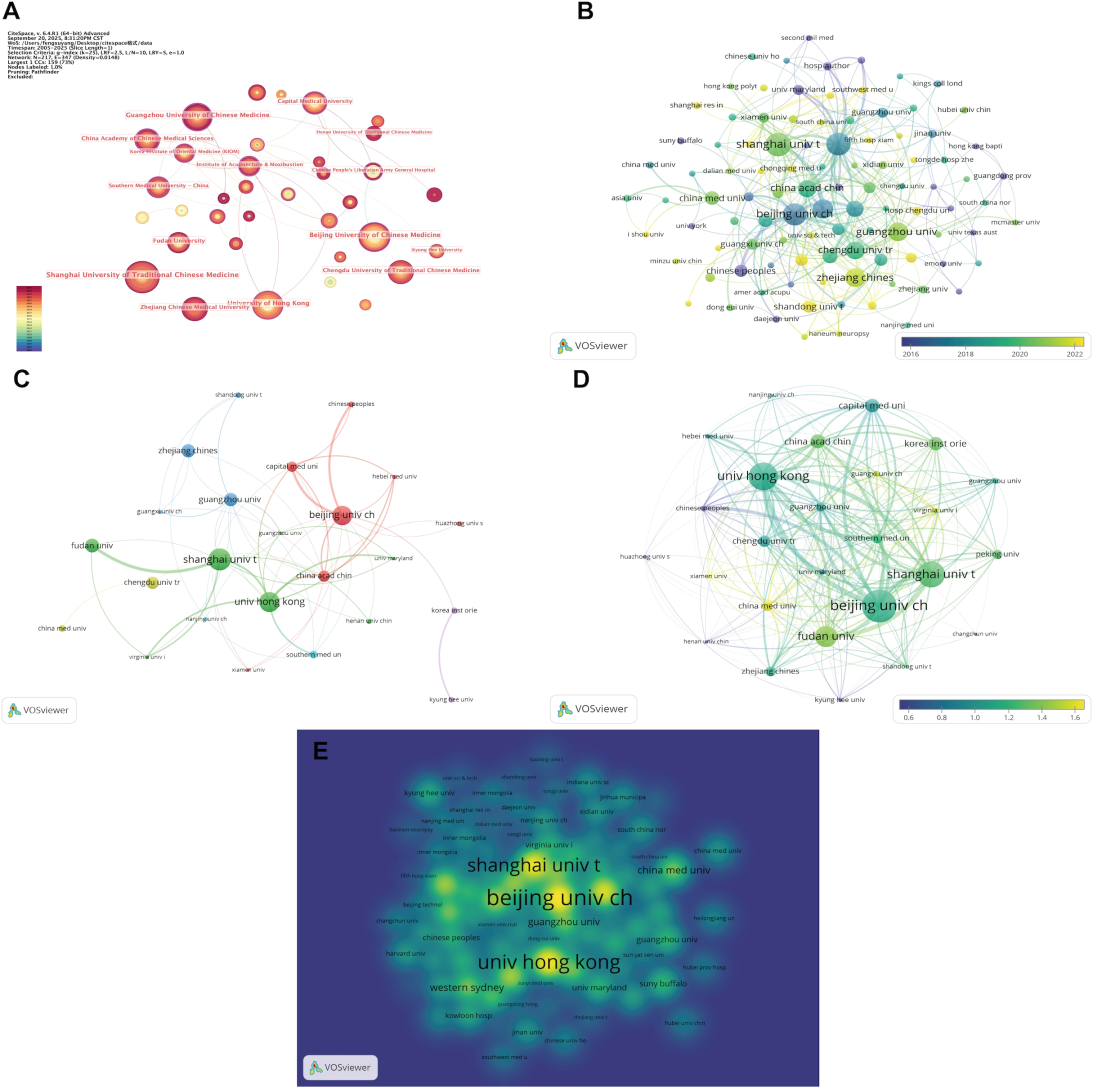
Institutional collaboration analysis. **(A)** Visualization of the institutional collaboration network. **(B)** Overlay visualization of institutional research activity. **(C)** Collaboration network among highly productive institutions (≥ 6 publications). **(D)** Overlay visualization of institutional citation impact. **(E)** Density visualization of institutional research intensity. (Node size represents the number of publications produced by each institution, with larger nodes indicating higher publication output. The thickness of the connecting lines reflects the strength of collaboration between institutions, with thicker lines indicating stronger collaborative relationships. The meaning of node colors varies across visualization types: in **(A, B)**, node color represents the average publication year, with warmer colors indicating more recent years; in **(C)**, node color denotes different clusters; in **(D)**, node color represents institutional impact, with warmer colors indicating greater influence; and in **(E)**, warmer colors indicate higher research intensity for the corresponding institution).

The institutional collaboration network consisted of 217 nodes and 347 links (**Figure 4A**). Shanghai University of Traditional Chinese Medicine, Beijing University of Chinese Medicine, and The University of Hong Kong were positioned at the core of the network and maintained high-intensity collaborations with various institutions.

The overlay visualization revealed that research activity in the past five years has primarily concentrated in the Yangtze River Delta and South China regions, including cities such as Shanghai, Zhejiang, Guangzhou, and Chengdu (**Figure 4B**). Among institutions with ≥ 6 publications, a major collaboration axis was formed between Shanghai University of Traditional Chinese Medicine, Fudan University, and The University of Hong Kong, while Beijing University of Chinese Medicine, the China Academy of Chinese Medical Sciences, and Capital Medical University constituted another key cluster. South Korea’s Kyung Hee University and the Korea Institute of Oriental Medicine also maintained steady cooperation (**Figure 4C**).

In terms of citation impact, institutions such as Xiamen University, Guangxi University of Chinese Medicine, and the University of Virginia demonstrated higher average citation intensity, reflecting their academic influence (**Figure 4D**). The density visualization further showed that research hotspots were concentrated around Beijing, Shanghai, and Hong Kong, followed by Guangzhou and selected nodes in Southwest China (**Figure 4E**).

**Table 4** presents the top 10 most productive institutions. Shanghai University of Traditional Chinese Medicine ranked first with 34 publications, followed by The University of Hong Kong (31 articles) and Beijing University of Chinese Medicine (30 articles). Notably, Beijing University of Chinese Medicine had the highest average citation rate (33 citations per article), indicating its strong academic impact.

**Table 4:** Top 10 most productive institutions.

Rank	Organization	Articles	Citations	Total link strength	Citations/article
1	Shanghai Univ Tradit Chinese Med	34	785	41	23
2	Univ Hong Kong	31	844	70	27
3	Beijing Univ Chinese Med	30	984	50	33
4	Fudan Univ	22	635	20	29
5	Guangzhou Univ Chinese Med	21	271	28	13
6	Zhejiang Chinese Med Univ	21	299	14	14
7	Chengdu Univ Tradit Chinese Med	19	350	18	18
8	China Acad Chinese Med Sci	18	419	32	23
9	Capital Med Univ	16	413	33	26
10	Southern Med Univ	12	280	12	23

### 3.4 Journal analysis

**Figure 5:**
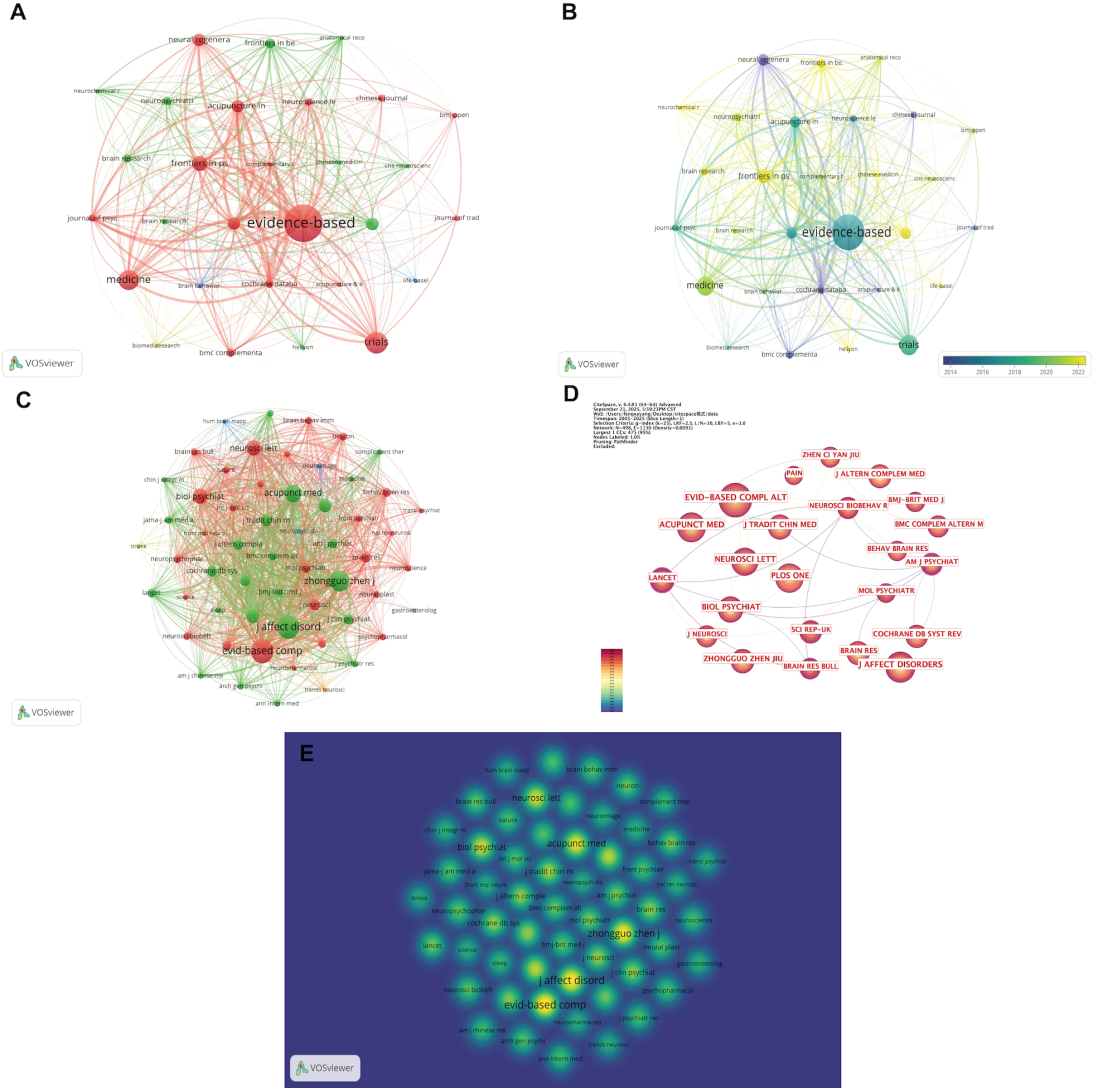
Journal analysis. **(A)** Visualization of the journal bibliographic coupling network. **(B)** Overlay visualization of journal bibliographic coupling. **(C)** Co-citation network of cited journals. **(D)** Journal citation relationship network. **(E)** Density visualization of highly cited journals. (Node size represents the number of publications or citation frequency of each journal, with larger nodes indicating higher publication output or greater citation counts. The thickness of the connecting lines reflects the strength of bibliographic coupling or co-citation between journals, with thicker lines indicating closer relationships. The meaning of node colors varies across visualization types: in **(A, C)**, node color represents cluster membership, with journals in the same cluster indicated by the same color; in **(B, D)**, node color represents the average publication year, with warmer colors indicating more recent years; and in **(E)**, warmer colors indicate higher citation levels for the corresponding journals).

The journal bibliographic coupling network consisted of 29 nodes and 356 links (**Figure 5A**).

The overlay visualization indicated that journals with high recent publication activity included Brain Research, Heliyon, and CNS Neuroscience & Therapeutics, suggesting a gradual expansion of research in this field toward neuroscience and interdisciplinary journals (**Figure 5B**).

Co-citation and citation network analyses of journals further demonstrated that Evidence-Based Complementary and Alternative Medicine and Acupuncture in Medicine served as key sources of publication and citation in this field, forming the core nodes of the citation landscape (**Figures 5C, D**).

The journal density visualization (**Figure 5E**) revealed that research hotspots were highly concentrated in core journals such as the Journal of Affective Disorders, Evidence-Based Complementary and Alternative Medicine, and Neuroscience Letters.

**Table 5** lists the top 13 most productive journals. Evidence-Based Complementary and Alternative Medicine ranked first with 29 publications and 589 citations, followed by Trials with 16 articles and 135 citations. Although the Cochrane Database of Systematic Reviews published only 6 articles, it achieved the highest citation count (438), indicating significant citation impact.

**Table 5:** Top 13 most productive journals.

Source	Articles	Citations	Citations/article	IF
Evidence-based Complementary and Alternative Medicine	29	589	20.3	2.65(Q3)
Trials	16	135	8.4	2.0(Q2)
Medicine	15	143	9.5	1.6(Q3)
Frontiers in Psychiatry	11	77	7.0	3.2(Q1)
Journal of Affective Disorders	9	269	29.9	4.9(Q1)
Acupuncture in Medicine	9	158	17.6	2.6(Q3)
Neural Regeneration Research	9	139	15.4	6.7(Q1)
Frontiers in Neuroscience	9	163	18.1	3.2(Q2)
Frontiers in Behavioral Neuroscience	7	55	7.9	2.9(Q2)
Cochrane Database of Systematic Reviews	6	438	73.0	9.4(Q1)
Brain Research Bulletin	6	106	17.7	3.7(Q2)
Neuropsychiatric Disease and Treatment	6	96	16.0	2.9(Q2)
BMC Complementary and Alternative Medicine	6	164	27.3	3.4(Q1)

Overall, research outputs in this field are primarily published in a limited number of mid- to high-impact journals related to integrative medicine, psychiatry, and neuroscience, reflecting a distinct interdisciplinary dissemination pattern.

### 3.5 Author analysis

**Figure 6:**
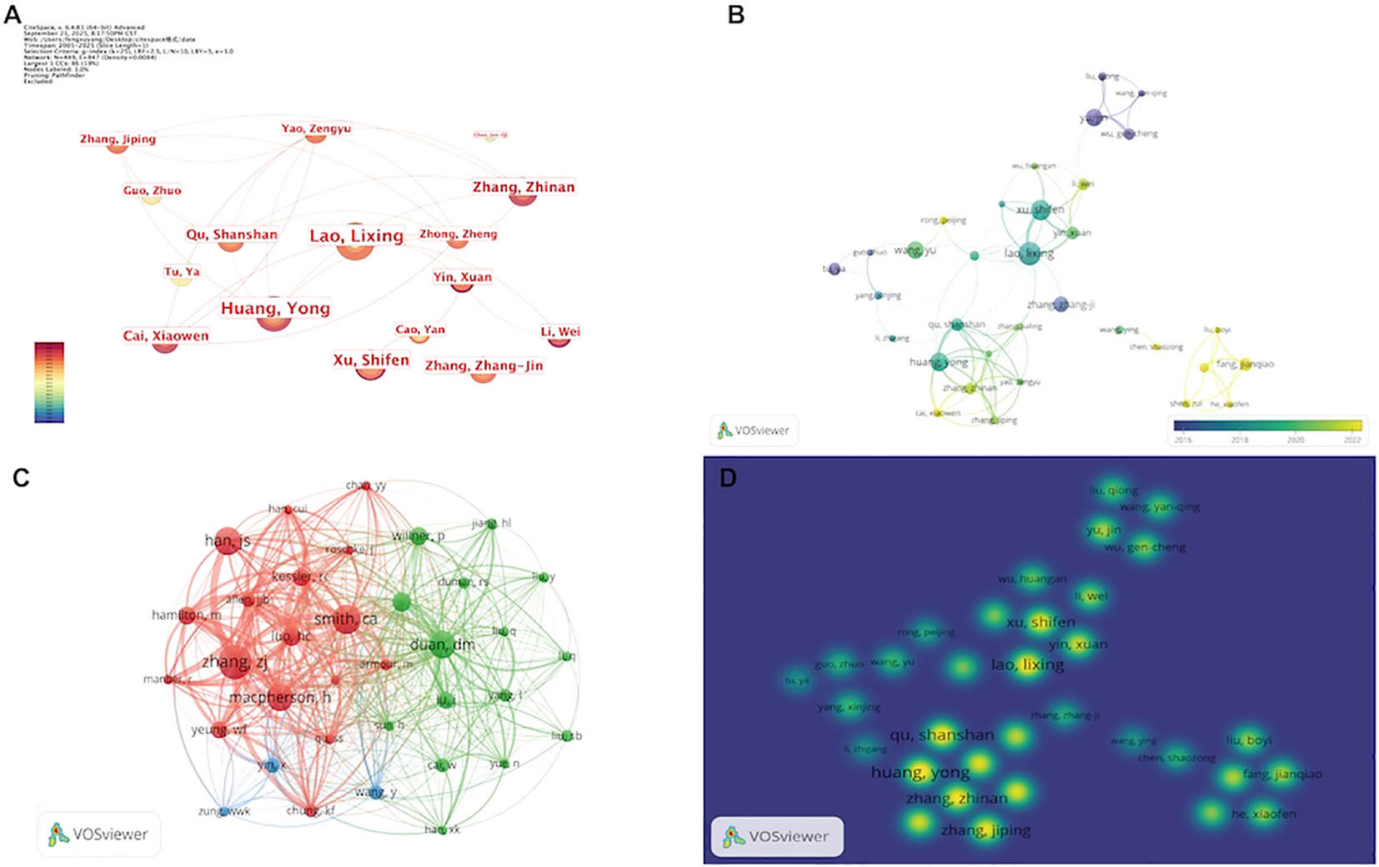
Author analysis. **(A)** Visualization of the author collaboration network. **(B)** Overlay visualization of author research activity. **(C)** Co-citation clustering network of cited authors. **(D)** Density visualization of author collaboration hotspots. (Node size represents the number of publications or citation frequency of each author, with larger nodes indicating higher publication output or greater citation counts. The thickness of the connecting lines reflects the strength of collaboration or co-citation between authors, with thicker lines indicating closer relationships. The meaning of node colors varies across visualization types: in **(A, B)**, node color represents the average publication year, with warmer colors indicating more recent years; in **(C)**, node color represents cluster membership, with authors in the same cluster indicated by the same color; and in **(D)**, warmer colors indicate higher levels of collaboration activity for the corresponding authors).

The author collaboration network consists of 449 nodes and 847 links (**Figure 6A**). The largest collaborative cluster is centered around Lao, Lixing, Xu, Shifen, and Huang, Yong, who maintain close connections with Yao, Zengyu, Yin, Xuan, and Cai, Xiaowen, among others.

Overlay visualization shows that authors such as Fang, Jianqiao and Liu, Boyi have emerged as active contributors in recent years, whereas Yu, Jin and Tu, Ya were among the early researchers in this field (**Figure 6B**).

The co-citation network of cited authors demonstrates a multi-cluster distribution. One cluster, represented by Smith, C.A., MacPherson, H., and Han, J.S., focuses primarily on clinical trials and evidence-based research. Another cluster, including Duan, D.M. and Liu, Q., is more engaged in mechanistic and neuroscientific studies (**Figure 6C**).

The density visualization indicates that research hotspots are concentrated around core teams led by Lao, Xu, Huang, and Zhang, while newly emerging high-density areas have formed around groups such as Fang, Jianqiao (**Figure 6D**).

**Table 6** presents the top 10 most productive authors in this field, along with their primary publishing journals and the corresponding impact factor (IF) quartiles. Lao, Lixing ranks first with 16 publications and 396 total citations, followed by Xu, Shifen (14 publications, 415 citations) and Huang, Yong (13 publications, 286 citations), indicating their substantial contributions to research on electroacupuncture for depression. Notably, Zhang, Zhang-jin has published 11 papers and exhibits the highest average citation rate (35.4 citations per article), suggesting a relatively high academic impact. In addition, Yin, Xuan has published in the high-impact journal JAMA Network Open (IF = 9.7), suggesting a relatively high academic visibility.

**Table 6:** Top 10 most productive authors.

Author	Articles	Citations	Citations/article	Main journal	IF (quartile)
Lao, Lixing	16	396	24.8	Evidence-based Complementary and Alternative Medicine	2.65(Q3)
Xu, Shifen	14	415	29.6	Nature and Science of Sleep	3.4(Q2)
Huang, Yong	13	286	22.0	Neural Regeneration Research	6.7(Q1)
Yu, Jin	12	317	26.4	Journal of Affective Disorders	4.9(Q1)
Wang, Yu	12	226	18.8	Evidence-based Complementary and Alternative Medicine	2.65(Q3)
Zhang, Zhang-jin	11	389	35.4	Neural Regeneration Research	6.7(Q1)
Fang, Jianqiao	9	133	14.8	CNS Neuroscience & Therapeutics	5.66(Q1)
Yin, Xuan	9	286	31.8	JAMA Network Open	9.7(Q1)
Qu, Shanshan	9	165	18.3	Neural Regeneration Research	6.7(Q1)
Tu, Ya	9	195	21.7	Neural Regeneration Research	6.7(Q1)

(Main journal refers to the journal in which each author published most frequently within the retrieved database. When multiple journals had the same number of publications, the journal with the highest impact factor was selected.).

### 3.6 Reference analysis

**Figure 7:**
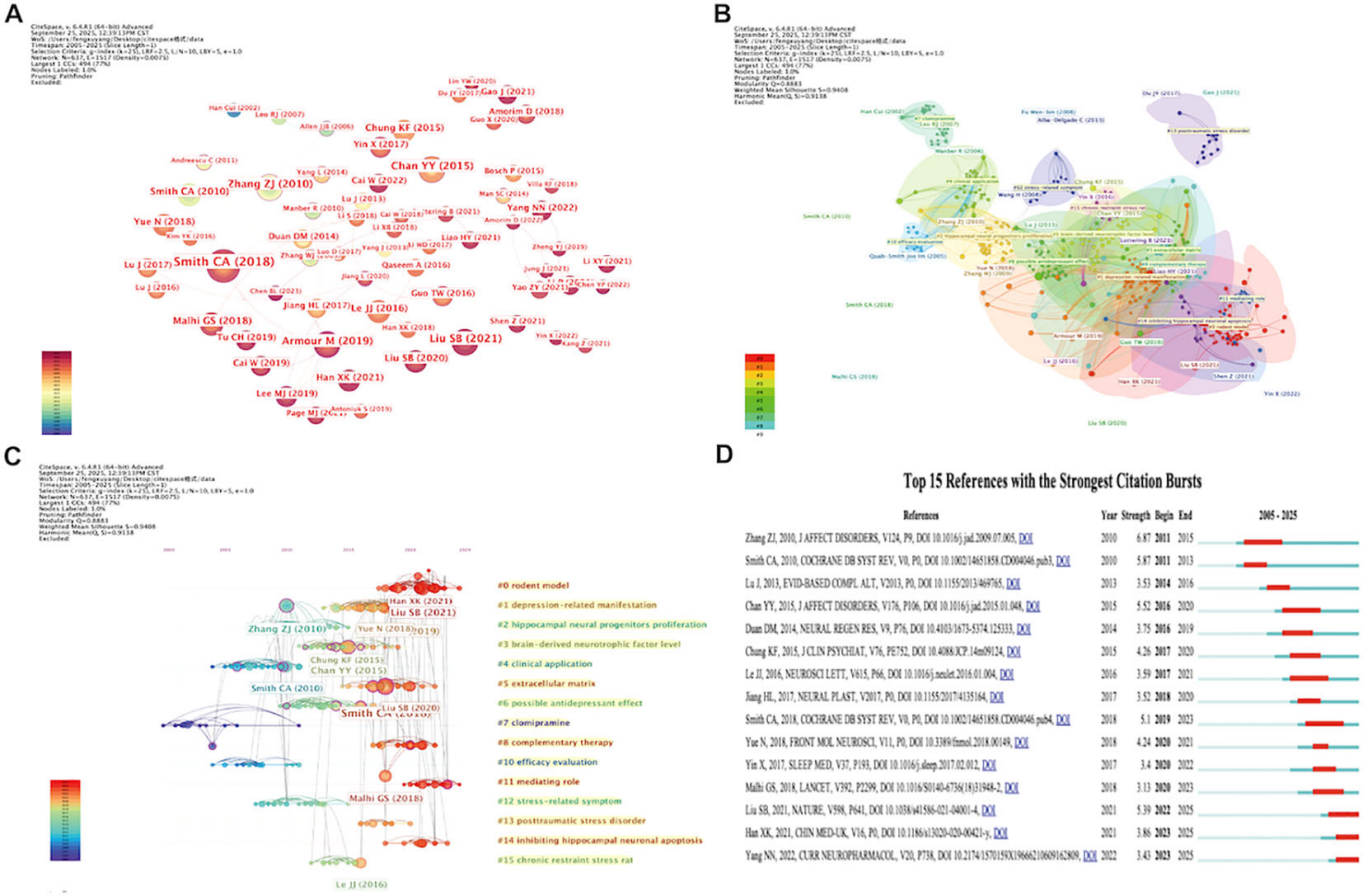
Reference analysis. **(A)** Visualization of the reference co-citation network. **(B)** Clustering visualization of reference co-citation. **(C)** Timeline visualization of co-citation clusters. **(D)** Burst detection visualization of the top 15 most cited references. (Node size represents the citation frequency of each reference, with larger nodes indicating higher citation counts. Node color denotes the publication year, with warmer colors corresponding to more recent years. The thickness of the connecting lines reflects the strength of co-citation between references, with thicker lines indicating stronger co-citation relationships. Notably, in **(B)**, node color represents cluster membership, with different colors indicating distinct clusters. In **(D)**, red line segments indicate the time periods during which references exhibit citation bursts, with higher values representing greater burst strength).

The reference co-citation network consists of 637 nodes and 1, 517 links (**Figure 7A**). The most frequently co-cited reference is the Hamilton Rating Scale for Depression (HAM-D) developed by Hamilton (1960) and published in the Journal of Neurology, Neurosurgery, and Psychiatry [doi:10.1136/jnnp.23.1.56], cited 32 times. This seminal work provides a critical methodological foundation for assessing depression severity (**Table 7**).

**Table 7:** Top 15 most highly cited references.

Rank	Count	Year	Cited references
1	32	1960	hamilton m, 1960, j neurol neurosur ps, v23, p56, doi 10.1136/jnnp.23.1.56
2	31	2010	zhang zj, 2010, j affect disorders, v124, p9, doi 10.1016/j.jad.2009.07.005
3	26	2018	smith ca, 2018, cochrane db syst rev, doi 10.1002/14651858.cd004046.pub4
4	25	1998	luo hc, 1998, psychiat clin neuros, v52, ps338, doi 10.1111/j.1440-1819.1998.tb03262.x
5	23	2003	han js, 2003, trends neurosci, v26, p17, doi 10.1016/s0166-2236(02)00006-1
6	21	2015	chan yy, 2015, j affect disorders, v176, p106, doi 10.1016/j.jad.2015.01.048
7	20	2000	röschke j, 2000, j affect disorders, v57, p73, doi 10.1016/s0165-0327(99)00061-0
8	20	2013	sun h, 2013, j altern complem med, v19, p733, doi 10.1089/acm.2011.0637
9	19	2013	qu ss, 2013, j psychiatr res, v47, p726, doi 10.1016/j.jpsychires.2013.02.004
10	19	2010	smith ca, 2010, cochrane db syst rev, doi 10.1002/14651858.cd004046.pub3
11	19	2016	le jj, 2016, neurosci lett, v615, p66, doi 10.1016/j.neulet.2016.01.004
12	18	2005	mukaino yoshito, 2005, acupunct med, v23, p70
13	18	2011	yeung wf, 2011, sleep, v34, p807, doi 10.5665/sleep.1056
14	18	1998	ulett ga, 1998, biol psychiat, v44, p129, doi 10.1016/s0006-3223(97)00394-6
15	17	2014	duan dm, 2014, neural regen res, v9, p76, doi 10.4103/1673-5374.125333

**Table 8:** Top 10 keywords in PubMed.

Rank	Keywords	Counts	Centrality
1	electroacupuncture	29	0.18
2	depression	24	0.18
3	model group	21	0.09
4	ea group	21	0.10
5	control group	18	0.39
6	depression rats	14	0.02
7	acupuncture	14	0.36
8	depressive symptoms	12	0.20
9	chronic unpredictable mild stress	12	0.34
10	depression model	11	0.18

Cluster analysis identified a total of 16 reference clusters. The largest cluster #0 “rodent model” primarily focuses on animal studies employing the chronic unpredictable mild stress (CUMS) model, highlighting the important role of animal models in mechanistic research. Several clusters are related to neurobiological mechanisms. For example, #2”hippocampal neural progenitors proliferation” and #3 “BDNF level” mainly reflect studies on neurogenesis and synaptic plasticity; #14 “inhibiting hippocampal neuronal apoptosis” emphasizes the role of inhibiting neuronal apoptosis in neuroprotection. In addition, #5”extracellular matrix”suggests that regulation of the brain microenvironment and structural remodeling is emerging as a new research direction. Clinical evidence–based clusters include #4”clinical application”, #10”efficacy evaluation”, and #8”complementary therapy”, which primarily reflect research directions related to clinical translation, efficacy assessment, and integrative therapeutic strategies (**Figure 7B**).

The timeline view shows that prior to 2010, co-cited literature mainly focused on efficacy evaluation, depression rating scales, and clinical intervention strategies. Since 2016, research has shifted progressively toward central nervous system mechanisms, especially regarding BDNF expression, hippocampal neuroplasticity, and stress-response modulation. This shift reflects the field’s transition from clinical outcome validation to in-depth mechanistic investigation (**Figure 7C**).

The top 15 references with the strongest citation bursts were identified, with burst strengths ranging from 3.13 to 6.87, most occurring after 2015. Among them, the most prominent and earliest burst was observed for the work by Zhang, Zhang-jin (2010) titled “The effectiveness and safety of acupuncture therapy in depressive disorders: Systematic review and meta-analysis” [doi:10.1016/j.jad.2009.07.005], with a burst period from 2010 to 2011. This study systematically evaluated the efficacy and safety of acupuncture (including electroacupuncture) for depression, providing a solid theoretical basis for subsequent research (**Figure 7D**).

### 3.7 Keyword analysis

**Figure 8:**
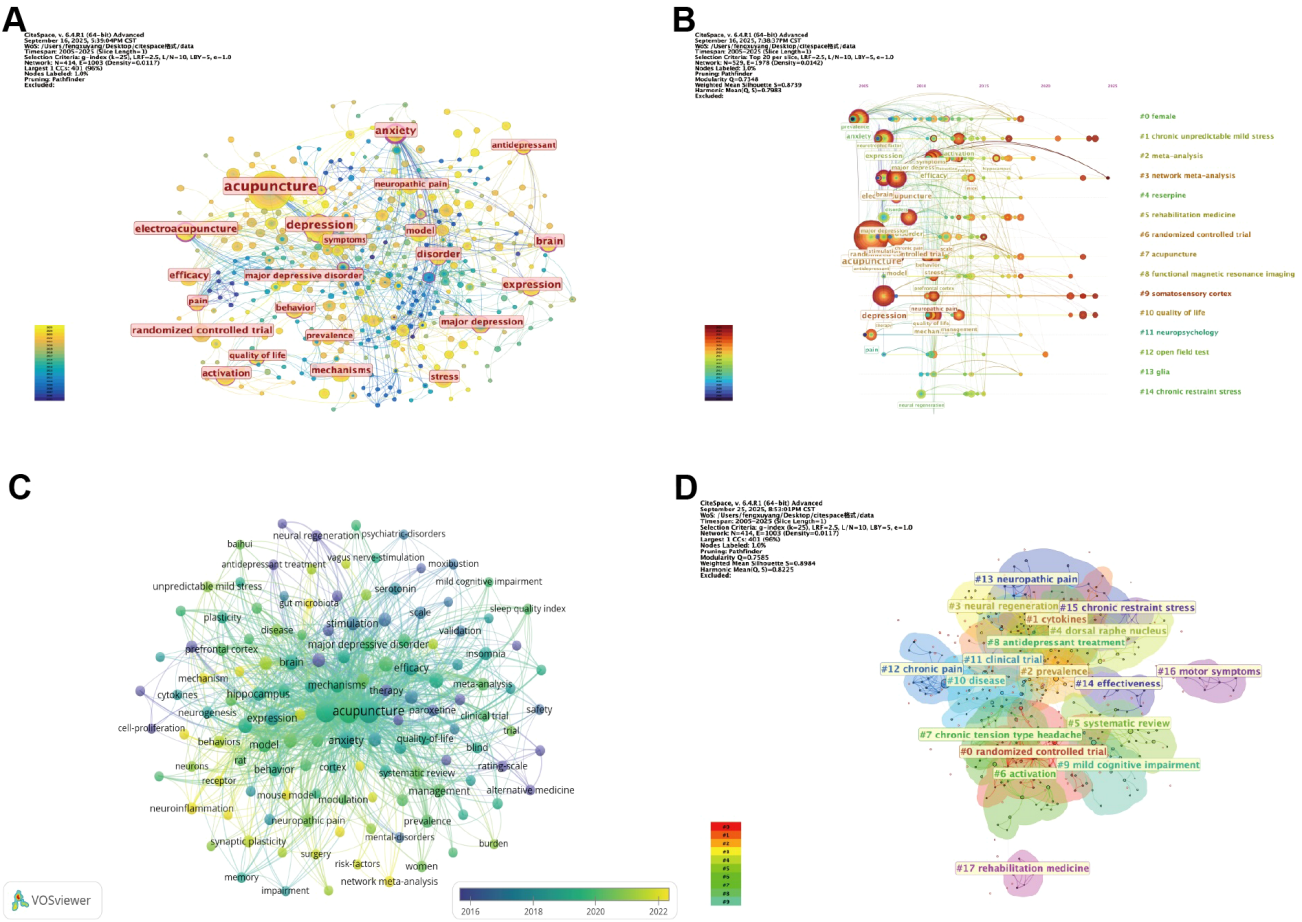
Keyword analysis. **(A)** Visualization of the keyword co-occurrence network. **(B)** Timeline visualization of keyword clusters. **(C)** Overlay visualization of keyword evolution. **(D)** Clustering visualization of the keyword co-occurrence network. (Node size represents the frequency of keyword occurrence, with larger nodes indicating higher occurrence frequency. Node color denotes the average publication year, with warmer colors corresponding to more recent years. The thickness of the connecting lines reflects the strength of co-occurrence between keywords, with thicker lines indicating closer relationships. Notably, in **(D)**, node color represents cluster membership, with different colors indicating distinct clusters).

The keyword co-occurrence network consists of 414 nodes and 1, 003 links (**Figure 8A**). Core terms such as “acupuncture, ” “depression, ” “anxiety, ” and “electroacupuncture” occupy central positions in the network with high connectivity.

The timeline view and overlay visualization reveal a shift in research focus over time. Earlier studies primarily emphasized clinical interventions and outcome evaluations, featuring keywords such as “randomized controlled trial, ” “efficacy, ” “pain, ” and “quality of life.” In recent years, research has expanded toward mechanistic dimensions, with frequent keywords including “meta-analysis, ” “network meta-analysis, ” “functional magnetic resonance imaging (fMRI), ” and “neuropsychology, ” reflecting a growing integration of evidence-based approaches and neuroscience (**Figures 8B, C**).

A total of 18 keyword clusters were identified through clustering analysis. The largest cluster #0, “randomized controlled trial” mainly reflects studies focusing on the clinical efficacy and safety evaluation of electroacupuncture for depression, highlighting the important role of randomized controlled trials in this field. In addition, cluster #1 “cytokines” mainly related to research themes involving inflammatory responses and immune regulation; cluster #3 “neurogenesis” reflects research themes related to neuroplasticity and neuronal regeneration; and cluster #8 “antidepressant treatment” suggests research directions involving the combined use of electroacupuncture with pharmacological or other therapeutic approaches. Clusters #7 “chronic tension-type headache”, #12 “chronic pain”, and #13 “neuropathic pain” reflect research on the comorbidity of depression and pain, suggesting growing interest in the application of electroacupuncture in pain-related emotional disorders (**Figure 8D**).

**Figure 9:**
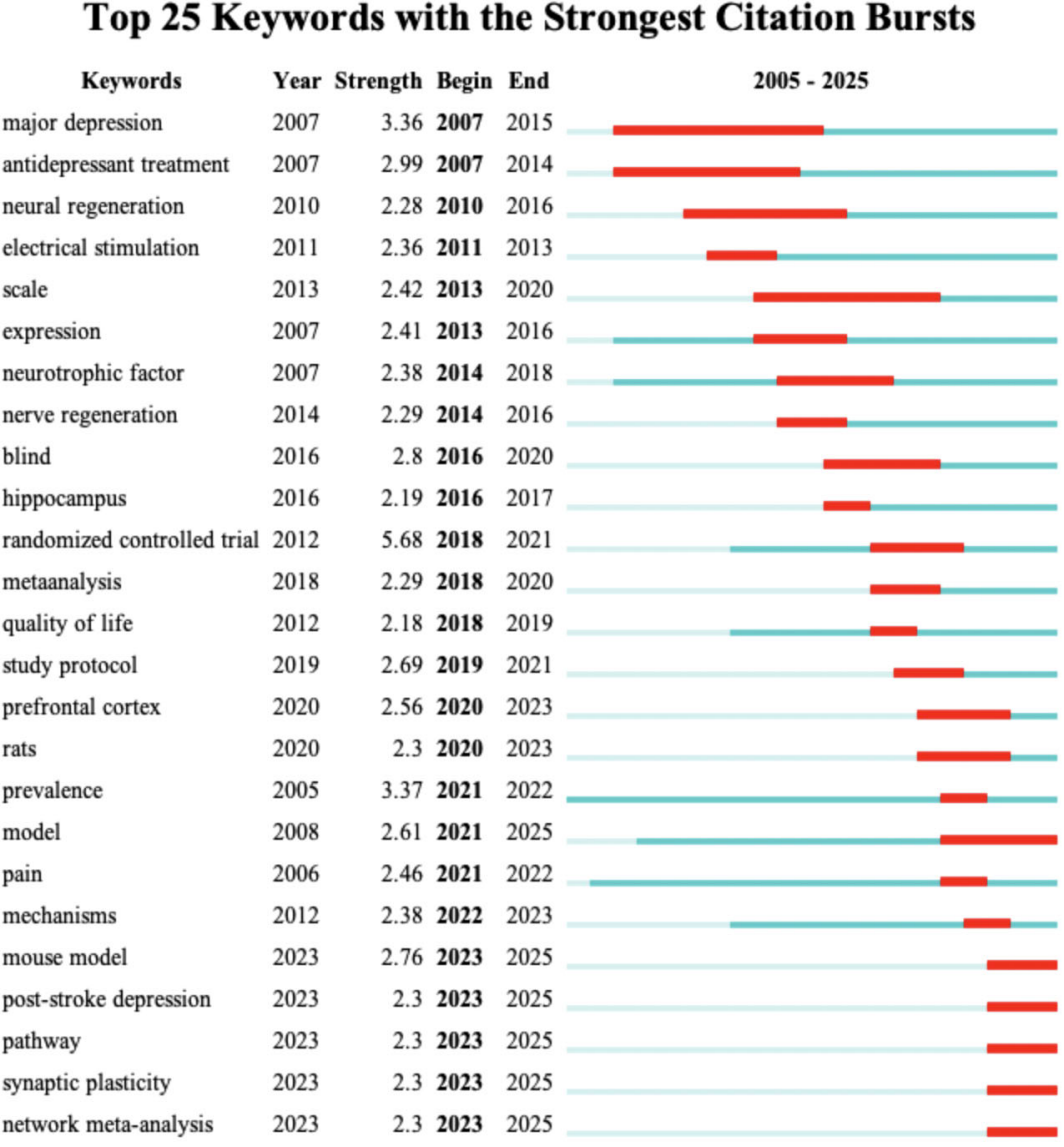
Top 25 keywords with the strongest citation bursts. (Red line segments indicate the time intervals during which the keyword exhibits citation bursts, with higher values representing greater burst strength).

Keyword burst detection identified 25 burst terms with burst strengths ranging from 2.18 to 5.68, spanning 2007 to 2025. In recent years, mechanism- and model-related terms have shown continuous citation bursts, such as “prefrontal cortex” (2.56, 2020–2023), “rats” (2.30, 2020–2023), “prevalence” (3.37, 2021–2022), “model” (2.61, 2021–2025), and “mechanisms” (2.38, 2022–2023). After 2023, terms including “mouse model” (2.76), “post-stroke depression” (2.30), “pathway” (2.30), “synaptic plasticity” (2.30), and “network meta-analysis” (2.30) emerged prominently, indicating the evolving research frontiers (**Figure 9**).

### 3.8 Supplementary comparative analysis using the PubMed database

PubMed, as a globally recognized high-quality biomedical database, contains a large number of authoritative medical publications. PubMed data were incorporated as a supplementary database for comparative analysis. Keyword analysis is an important approach for identifying research themes and emerging frontiers. Therefore, co-occurrence, clustering, burst detection, and timeline analyses were conducted on PubMed keywords, and comparative analyses of keyword trends between PubMed and the WoSCC were performed to evaluate their consistency and complementarity.

**Figure 10:**
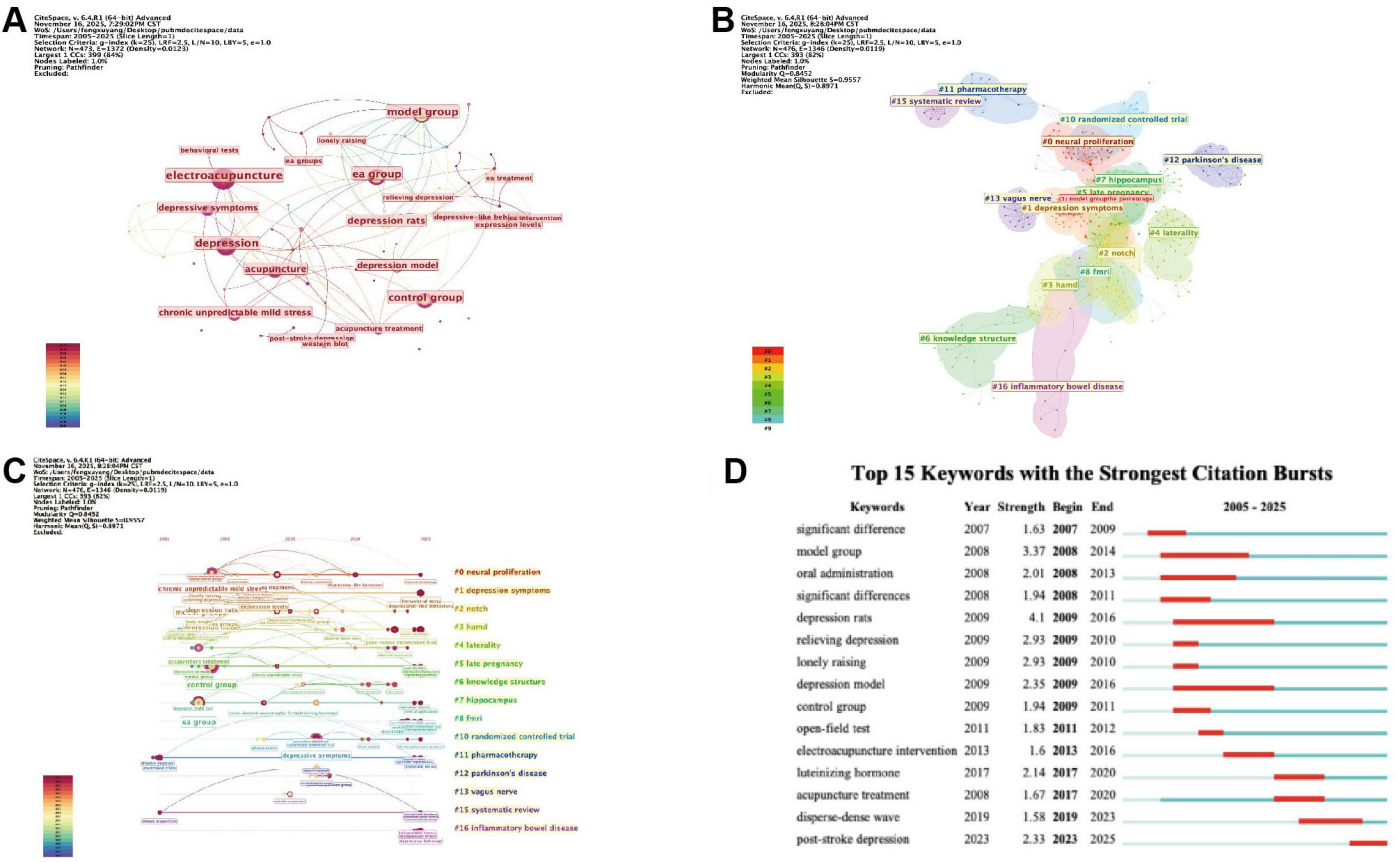
Keyword analysis using the PubMed database. **(A)** Visualization of the PubMed keyword co-occurrence network. **(B)** Clustering visualization of the PubMed keyword co-occurrence network. **(C)** Timeline visualization of PubMed keyword clusters. **(D)** Burst detection visualization of the top 15 keywords with the strongest citation bursts in PubMed. (Node size represents the frequency of keyword occurrence, with larger nodes indicating higher occurrence frequency. Node color denotes the average publication year, with warmer colors corresponding to more recent years. The thickness of the connecting lines reflects the strength of co-occurrence between keywords, with thicker lines indicating closer relationships. Notably, in **(10B)**, node color represents cluster membership, with different colors indicating distinct clusters. In **(10D)**, red line segments indicate the time intervals during which keywords exhibit citation bursts, with higher values representing greater burst strength).

**Figure 11:**
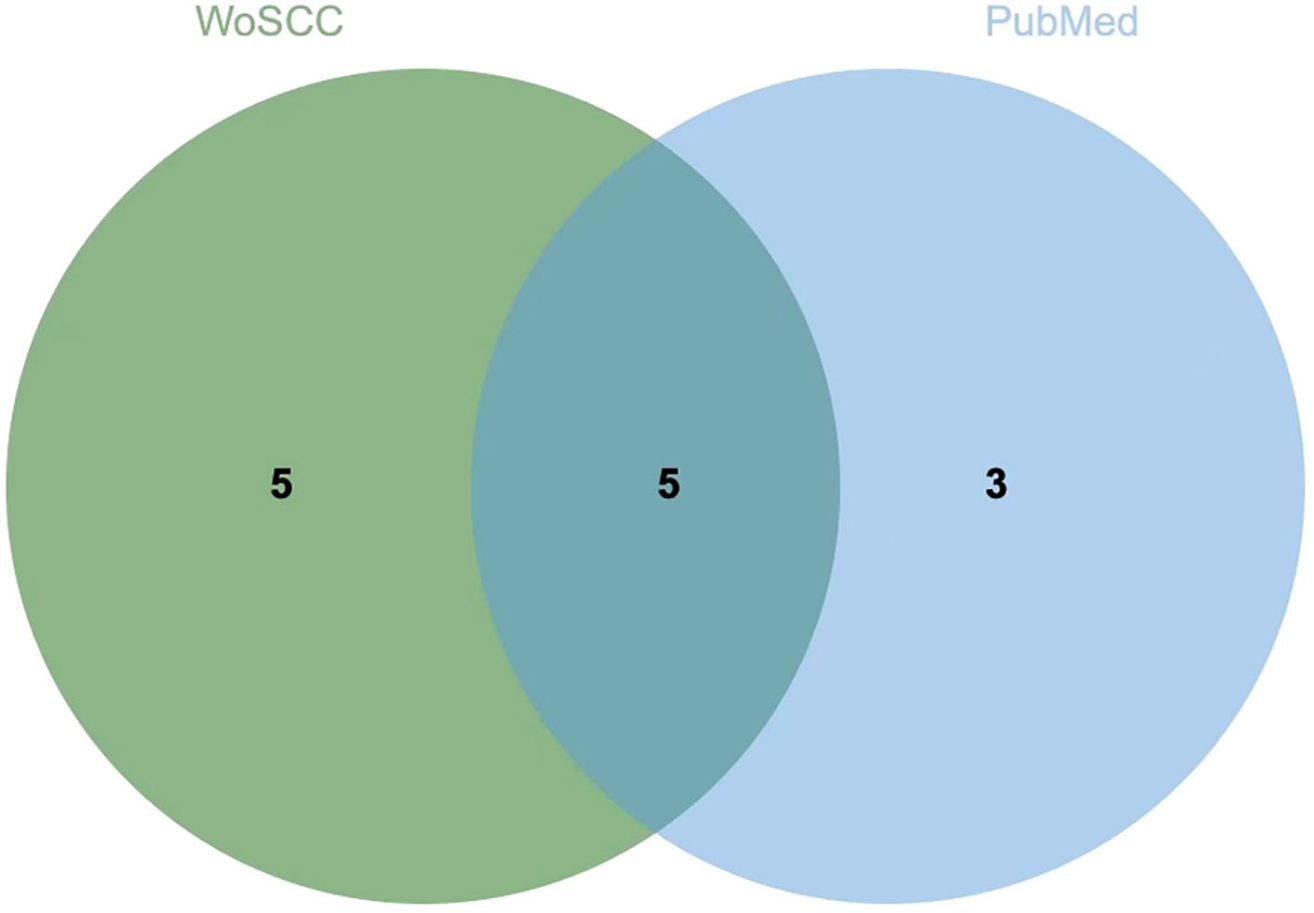
Venn diagram of high-frequency keywords between WoSCC and PubMed. (To improve the validity of cross-database comparison, non-thematic or overly generic terms were excluded, while representative mechanism-related terms (e.g., western blot) were retained to reflect experimental research trends).

The keyword co-occurrence network consisted of 473 nodes and 1, 372 links (**Figure 10A**). Core terms such as “acupuncture, ” “electroacupuncture, ” “depression, ” “chronic unpredictable mild stress, ” and “depressive symptoms” occupied central positions in the network. In the comparison of high-frequency keywords between the two databases, five overlapping keywords (5 of 13, approximately 38%) were identified, including “electroacupuncture, ” “depression, ” “acupuncture, ” “randomized controlled trial, ” and “post-stroke depression, ” indicating a certain level of consistency in core topics between WoSCC and PubMed. Meanwhile, each database also exhibited unique keywords (WoSCC: anxiety, brain, mechanism, expression, behavior; PubMed: chronic unpredictable mild stress, western blot) (**Figure 11**, **Table 8**, **Supplementary Material 1**).

Clustering analysis identified a total of 17 keyword clusters. Among them, cluster #0 “neurogenesis”, cluster #1 “depressive symptoms”, and cluster #10 “randomized controlled trial” were highly consistent with the clustering results of the WoSCC, corresponding to research themes related to neuroplasticity, clinical symptom assessment, and study design, respectively. In addition, the PubMed revealed several distinct research themes: cluster #7 “hippocampus” and cluster #8 “fMRI” reflect the application of specific brain regions and neuroimaging techniques, suggesting an increasing use of neuroscience-related approaches in this field; cluster #5 “late pregnancy” indicates growing attention to specific populations, particularly perinatal depression (**Figure 10B**).

Timeline analysis of keyword evolution showed that research focus has gradually shifted from clinically oriented studies to themes related to neural and molecular research. Early studies were dominated by keywords such as “effective treatment,” “controlled trial,” and “clinical observation,” whereas in recent years, topics such as “protein expression,” “signaling pathway,” and “neuronal morphology” have become increasingly prominent. This trend is consistent with observations from the WoSCC (**Figure 10C**).

Burst detection identified a total of 15 burst keywords, with burst strengths ranging from 1.58 to 3.37, spanning from 2007 to 2025. Compared with WoSCC, differences were observed in the distribution of emerging research hotspots. Recent burst keywords in WoSCC, including “mouse model,” “pathway,” “synaptic plasticity,” and “network meta-analysis,” mainly reflect directions related to preclinical experimental studies, molecular and neuro-related research themes, and methodological development. In contrast, PubMed highlighted keywords such as “dense-sparse wave,” “acupuncture treatment,” and “luteinizing hormone,” reflecting differences in focus on intervention patterns, physiological indicators, and specific research directions. Notably, “post-stroke depression” emerged as a burst keyword in both databases, indicating its importance as a current research frontier.

Overall, WoSCC and PubMed show consistency in core research themes, while differences in emerging hotspots highlight their complementary strengths (**Figure 10D**).

## 4 Discussion

### 4.1 Major findings

This study conducted a bibliometric analysis of 317 articles on electroacupuncture for depression published between 2005 and 2025 in the WoSCC, involving 22 countries/regions, 375 institutions, 127 journals, and 1, 825 authors. The results indicate that while depression and related affective disorders had become key research topics as early as the beginning of the 21st century, systematic studies on electroacupuncture as an intervention method emerged relatively late. From 2005 to 2013, the annual number of publications was low, but a significant increase has been observed since 2017, reflecting the growing academic attention to electroacupuncture as a potential therapeutic approach for depression.

In terms of geographic distribution, China was the leading contributor, followed by the United States and South Korea, jointly forming the primary research force in this field—a pattern closely tied to the strong clinical foundations of acupuncture and traditional medicine in these countries. Regarding international collaboration, China and the United States maintained the closest and most stable cooperation, with China occupying a central hub position in the collaboration network and remaining highly active over the past five years. Although China ranks first in terms of publication volume in this field, the average citations per article remain relatively low, suggesting that the overall research impact still needs to be improved. Several factors may account for this phenomenon. First, based on the collaboration network analysis, research collaborations are primarily concentrated among institutions in China. Although China–USA collaborations appear relatively frequent, the overall level of international collaboration remains limited, which may, to some extent, influence the international dissemination and citation of the related research. Second, from the perspective of research types, studies in this field are, to some extent, dominated by basic research, while clinical translational studies remain relatively limited, which may limit the application and impact of research findings in the international clinical context. Third, from the perspective of journal distribution, a large proportion of studies are published in journals with relatively low impact factors or limited international visibility, which may reduce their academic visibility and consequently affect citation frequency. At the institutional level, Shanghai University of Traditional Chinese Medicine, Beijing University of Chinese Medicine, and the University of Hong Kong served as key cooperation hubs, maintaining stable partnerships with South Korea. Recent research activity and density hotspots were concentrated in the Yangtze River Delta and South China regions, reflecting their comprehensive advantages in clinical resources, interdisciplinary platforms, and international publication capabilities.

The major publishing journals were mostly core journals in the fields of evidence-based medicine and neuropsychiatry with moderate impact, suggesting that the recognition of electroacupuncture in top-tier mainstream medical journals still needs to be improved. Among authors, Lao Lixing from China published the most articles. The most cited reference was the Hamilton Depression Rating Scale (HAM-D) by Hamilton M, which, although not specific to electroacupuncture, has been widely used in efficacy evaluations and comparative studies due to its role as a standardized outcome measure for depression.

Keyword analysis indicates that research focus has shifted from the core cluster of “depression–electroacupuncture–clinical outcomes” toward two main directions: “evidence-based evaluation” and research themes related to “neuroimaging, neuroplasticity, and glial-related processes, ” reflecting increasing attention to neural circuit- and glial cell–related studies. In addition, supplementary analysis based on the PubMed database shows that clinical interventions and randomized controlled trials remain core research themes in electroacupuncture for depression, with recent studies exhibiting a shift from efficacy validation toward neuro-related research themes.

### 4.2 Main mechanistic research themes of electroacupuncture for depression

Based on the bibliometric analysis, this study identified several representative mechanism-related research themes in this field. An overview of these themes is provided in conjunction with the relevant literature to facilitate understanding of the research hotspots and trends reflected by the bibliometric results. It should be noted that these themes primarily reflect the current focus of research and do not represent well-established, definitive mechanisms of action.

#### 4.2.1 Research themes related to neurogenesis and synaptic plasticity associated with the BDNF signaling pathway

Neurogenesis refers to the process by which neural stem cells or progenitor cells differentiate into mature neurons. In the adult brain, the dentate gyrus of the hippocampus and the subventricular zone retain a limited capacity for neurogenesis, which is essential for learning, memory, and emotional regulation (19, 20). Synaptic plasticity refers to the ability of synapses to modify their transmission efficiency and serves as the foundation for learning and memory. Studies have shown that hippocampal neuroplasticity is significantly reduced in depressive animal models, accompanied by the downregulation of related protein expression (21).

BDNF is a key regulator of neurogenesis and synaptic plasticity and is considered to be closely associated with the development of depression. Studies have reported that decreased BDNF levels in the hippocampus of patients with depression may be accompanied by inhibited proliferation of neural progenitor cells, impaired neuronal differentiation, and disrupted synaptic stability (22, 23). Electroacupuncture may upregulate BDNF expression, thereby influencing these processes to some extent and improving depressive states (24–26).

The BDNF/TrkB/CREB signaling pathway has been suggested to be associated with these effects. TrkB (tropomyosin receptor kinase B), a high-affinity receptor of BDNF, possesses tyrosine kinase activity and can activate downstream signaling cascades, including the MAPK/ERK and PI3K/Akt pathways. The MAPK/ERK pathway phosphorylates CREB (cAMP response element-binding protein), which subsequently translocates into the nucleus to promote the transcription of depression-related genes, thereby forming a positive feedback loop (BDNF–TrkB–CREB) that participates in enhancing neurogenesis, neuronal differentiation, and synaptic stabilization (27–29).

In addition, electroacupuncture may also activate the Nrf2/HO-1 antioxidant pathway through the upregulation of BDNF. Nuclear factor erythroid 2-related factor 2 (Nrf2), as a key regulator of antioxidant defense, promotes the expression of heme oxygenase-1 (HO-1), reduces damage induced by reactive oxygen species (ROS), maintains cellular homeostasis, protects neurons, and improves depression (30, 31).

#### 4.2.2 Research themes related to the inhibition of inflammatory responses and glial cell overactivation

The “inflammatory response model” of depression suggests that abnormal activation of immune-inflammatory pathways may be involved in the development of depression. Peripheral pro-inflammatory cytokines (such as IL-6 and TNF-α) can cross the blood–brain barrier and are associated with alterations in neurotransmitter metabolism and neuroplasticity, thereby influencing the development of depression (32). The NLRP3 inflammasome, as a key inflammatory complex, can activate Caspase-1 and promote the maturation and release of IL-1β and IL-18, which are upregulated in emotion-related brain regions such as the hippocampus and prefrontal cortex (33, 34). Studies suggest that electroacupuncture may be associated with the regulation of the NF-κB/NLRP3 pathway, reduction of inflammasome activation, and downregulation of IL-1β, IL-6, and TNF-α expression, thereby potentially alleviating neuroinflammation and depressive-like behaviors (35).

Glial cells, particularly microglia and astrocytes, represent another important focus of electroacupuncture-related research. Under chronic stress, microglia may shift toward a pro-inflammatory M1 phenotype, releasing cytokines and neurotoxic mediators, which exacerbate neuronal damage and depressive symptoms (36–38). Studies suggest that electroacupuncture may inhibit M1 polarization while promoting anti-inflammatory M2 transformation, thereby potentially reducing inflammatory cytokine levels and protecting neuronal integrity (39, 40).

In depressive states, astrocytes become overactivated and are involved in glial scar formation, synaptic dysfunction, and impaired BDNF secretion (41–43). Existing evidence suggests that electroacupuncture may regulate GFAP expression, inhibit astrocyte overactivation, and stabilize neuron–synapse homeostasis, thereby improving depressive behaviors (44, 45).

#### 4.2.3 Research themes related to the regulation of the prefrontal cortex–amygdala/hippocampus circuit

The prefrontal cortex (PFC) is a key region governing emotion and cognition, while the amygdala primarily processes fear and negative emotions. Under normal conditions, the PFC inhibits amygdala hyperactivity to maintain emotional balance. In depression, the PFC–amygdala circuitry becomes dysregulated, with reduced PFC activity and amygdala overactivation, leading to abnormal emotional processing (46–50). Functional MRI (fMRI) studies have revealed disrupted connectivity between the amygdala and the left rostral PFC, correlating with negative affect and depressive symptoms. Studies suggest that EA may enhance prefrontal regulation of emotional processing and suppress amygdala hyperactivation, potentially improving depressive symptoms (51, 52).Additionally, existing evidence suggests that EA may restore E/I (excitatory/inhibitory) balance in pyramidal neurons of the dlPFC, mPFC, and hippocampal CA1, potentially helping to rebalance neural networks and alleviate anxiety- and depression-like behaviors (53).

#### 4.2.4 Research themes related to the regulation of HPA axis dysfunction

The hypothalamic–pituitary–adrenal (HPA) axis is a critical neuroendocrine system mediating stress responses. Chronic stress leads to continuous HPA activation, elevated levels of CRH, ACTH, and cortisol, and impaired negative feedback, thereby disturbing neurotransmission, inhibiting neurogenesis and promoting inflammation; these alterations are considered to be closely associated with the development and progression of depression (54). Studies suggest that EA may inhibit excessive activation of NMDA receptors (especially the GluN2A subunit) in the hypothalamus, potentially blocking downstream ERK/CREB phosphorylation, downregulating CRH expression, and restoring HPA homeostasis (28). Transcriptomic analyses further reveal that EA reverses differential gene expression in the hypothalamus of depression model rats, normalizing 78 genes associated with neuroregulation, hormone synthesis, and inflammation, suggesting that EA may remodel the hypothalamic transcriptome to correct HPA dysregulation (55).

### 4.3 Research hotspots and future trends

In recent years, research on EA for depression has increased markedly, consistent with the trend toward evidence-based and standardized development of traditional Chinese therapies (56–58). China remains the leading contributor due to its robust TCM foundation, with the United States and South Korea also playing important roles in international dissemination (59–61). The research focus has evolved from clinical efficacy validation to mechanistic elucidation, extending from intervention outcomes to neurobiological underpinnings (7, 62).

Keyword clustering and burst analyses indicate a transition from early clinical-outcome studies (“randomized controlled trial, ” “efficacy, ” “pain, ” “quality of life”) to mechanistic explorations involving “neurogenesis, ” “hippocampus, ” “inflammation, ” and “synaptic plasticity” (63, 64). The rise of terms such as “prefrontal cortex, ” “glia, ” and “gut microbiota” further suggests growing attention to EA’s modulation of neural networks, neurotrophic and inflammatory factors, and the microbiota–gut–brain axis (52, 65–69).

Meanwhile, advances in multimodal technologies—including fMRI, EEG, metabolomics, and neuroimaging—have provided objective molecular and visual evidence supporting EA’s antidepressant mechanisms (70–75). The integration of neuropsychological assessments, CUMS models, and gut–brain axis frameworks demonstrates that animal studies continue to play a crucial role in elucidating neural circuit regulation by EA (44, 76–80).

Overall, this field is transitioning from traditional clinical validation toward mechanistic and multimodal integration, forming an interdisciplinary framework linking neuroscience, psychiatry, molecular biology, and acupuncture theory. Understanding these hotspots and trends will help guide future translational and clinical research on EA for emotional disorders.

### 4.4 Limitations

This study has several limitations. First, although the analysis was primarily based on the Web of Science Core Collection and supplemented with PubMed data, non-English and regional databases (e.g., CNKI, Wanfang, and VIP) were not systematically searched, which may have resulted in the omission of regionally significant studies. In addition, the Web of Science database is known to have a bias toward English-language publications, which may further affect the comprehensiveness of the literature retrieval. Second, due to the constraints of the search time frame and cutoff date, some earlier or more recently published studies may not have been included. Third, this study primarily relied on quantitative bibliometric indicators, such as publication counts and citation frequencies. These metrics mainly reflect research activity and academic attention rather than trial quality, mechanistic validity, or clinical efficacy. In addition, this study did not systematically assess the methodological quality of the included studies, which may have led to an overemphasis on highly cited studies with less rigorous designs, while high-quality studies with lower citation counts may have been underrepresented. Fourth, keyword co-occurrence and clustering analyses inherently involve a certain degree of subjectivity. The results may be influenced by the choice of analytical tools, clustering algorithms, and threshold settings, potentially leading to variability in the findings; therefore, the conclusions should be interpreted with caution. Finally, the use of multiple visualization tools may have led to minor inconsistencies in figure styles and annotations.

## 5 Conclusion

This bibliometric and visual analysis provides a comprehensive overview of the international research landscape, thematic hotspots, and major mechanism-related research themes of electroacupuncture for depression. Although research in this field continues to expand, greater international collaboration, methodological rigor, and interdisciplinary integration are crucial to enhance research quality and promote research development. Strengthening these aspects will further improve the research level and international influence of electroacupuncture in the field of emotional disorders.

## Data Availability

The original contributions presented in the study are included in the article/**Supplementary Material**. Further inquiries can be directed to the corresponding authors.
