# Supplementary material for: Research trends and hotspots of electroacupuncture for depression: a bibliometric and visual analysis (2005–2025)
Source: Front Psychiatry. 2026 Apr 28;17:1749844. doi: 10.3389/fpsyt.2026.1749844 (PMC13161169; doi:10.3389/fpsyt.2026.1749844)
Supplement: Supplementary file 1 [file DataSheet1.docx]

Supplement1：
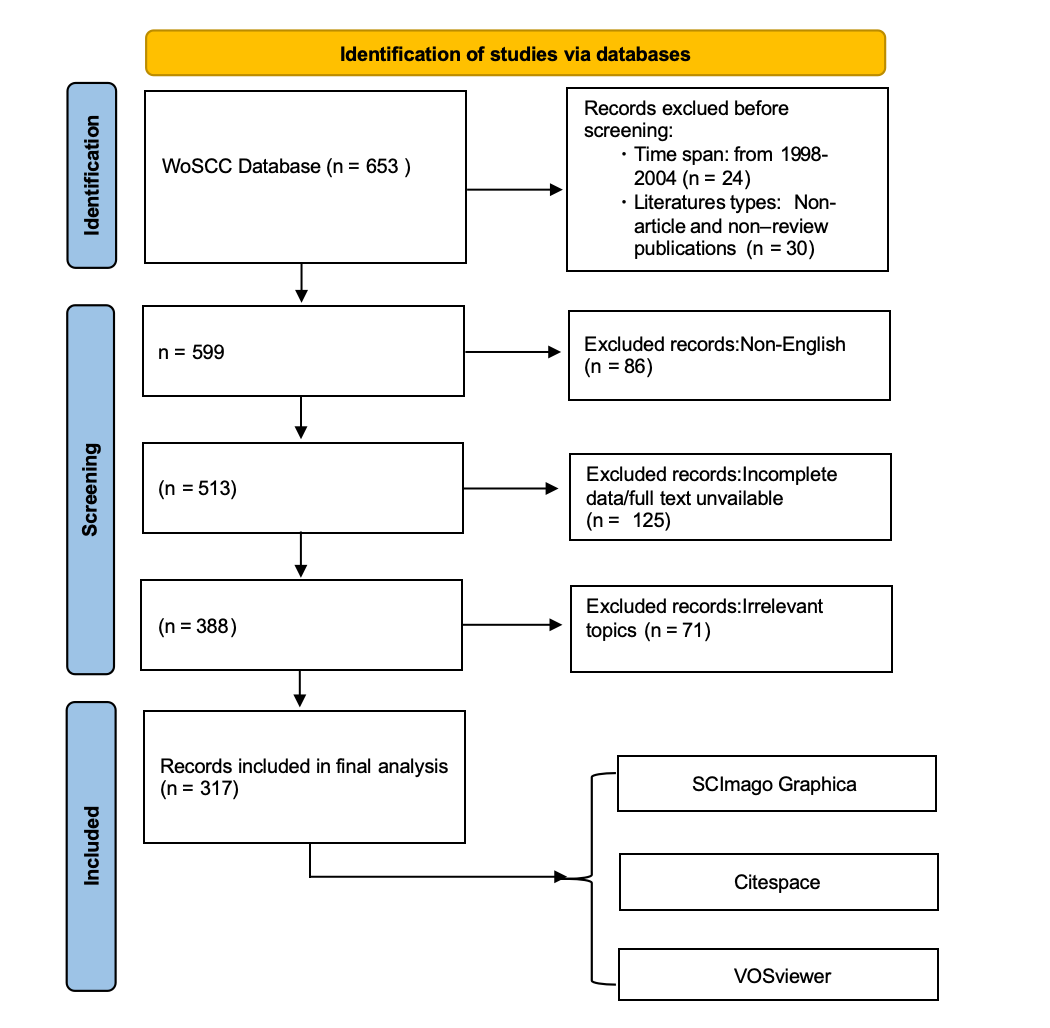


Supplementary Figure 1. The PRISMA flow diagram.

| Category | Keywords |
| --- | --- |
| WoSCC only | anxiety;meta-analysis;systematic review;chronic pain;hippocampus |
| Overlap | electroacupuncture;acupuncture;depression;randomized controlled trial;post-stroke depression |
| PubMed only | chronic unpredictable mild stress;depression symptoms;western blot |

Supplementary Table 11. Comparison of high-frequency keywords identified from WoSCC and PubMed using database-specific thresholds.
